# Cell cycle regulators p27 and pRb in lymphomas – correlation with histology and proliferative activity

**DOI:** 10.1054/bjoc.2000.1435

**Published:** 2000-11

**Authors:** M Kiviniemi, I Sauroja, A Rajamäki, K Punnonen, K-O Söderström, E Salminen

**Affiliations:** Department of Clinical Chemistry, University of Turku, Turku, 20014; MediCity Research Laboratory, University of Turku, Tykistökatu 6 A, 4th Floor, Turku, 20520; Turku Graduate School of Biomedical Sciences, University of Turku, Turku, 20014; Department of Hematology, University of Turku, Turku, 20014,; Department of Clinical Chemistry, University of Kuopio, PO Box 1777, Kuopio, 70211; Department of Pathology, University of Turku, Turku, 20014; Department of Oncology and Radiotherapy, University of Turku, Turku, 20014, Finland

**Keywords:** non-Hodgkin’s lymphoma, p27 (Kip1), pRb, Ki-67 (MIB-1)

## Abstract

The cell cycle is a complex event in which multiple regulator-proteins participate. The G _1_/S checkpoint of the cell cycle is controlled by pRb protein, which functions in its hypophosphorylated form as a negative regulator of growth. p27 (Kip1), a member of CIP/KIP family of cyclin inhibitory proteins, participates in inhibition of forming complexes that allow pRb to phosphorylate and lead the cell into mitosis. The expression of these important cell cycle regulator proteins was studied in a total of 96 non-Hodgkin’s lymphoma (NHL) samples, which were classified according to the REAL classification. The expression of p27, pRb and the cell proliferation marker Ki-67 (MIB-1) was evaluated in lymphomas using immunohistochemistry. This study showed that there were coordinate changes in the expression of p27 and pRb in NHL. When compared to low-grade lymphomas, high-grade lymphomas showed significantly reduced expression of p27 and inversely pRb expression was increased (*P* < 0.001). Increase in expression of Ki-67 was parallel with pRb expression, and was mainly seen in cells that lacked p27 expression (*P* < 0.0001). This study suggests that changes in the control of the cell cycle closely relate to the pathobiology of NHL. © 2000 Cancer Research Campaign


British Journal of Cancer (2000) 83(9), 1161–1167
© 2000 Cancer Research Campaign
doi: 10.1054/ bjoc.2000.1435, available online at http://www.idealibrary.com on IDEAL®

### Cell cycle regulators p27 and pRb in lymphomas – correlation with histology and proliferative activity

**M Kiviniemi[1,2,3], I Sauroja[2,3], A Rajamäki[4], K Punnonen[5], K-O Söderström[6] and E Salminen[7]**

[1]Department of Clinical Chemistry, University of Turku, 20014 Turku; [2]MediCity Research Laboratory, University of Turku, Tykistökatu 6 A, 4th Floor, 20520 Turku; [3]Turku Graduate School of Biomedical Sciences, University of Turku, 20014 Turku; [4]Department of Hematology, University of Turku, 20014 Turku; [5]Department of Clinical Chemistry, University of Kuopio, PO Box 1777, 70211 Kuopio; [6]Department of Pathology, University of Turku, 20014 Turku; [7]Department of Oncology and Radiotherapy, University of Turku, 20014 Turku, Finland

**Summary** The cell cycle is a complex event in which multiple regulator-proteins participate. The $G_1$/S checkpoint of the cell cycle is controlled by pRb protein, which functions in its hypophosphorylated form as a negative regulator of growth. p27 (Kip1), a member of CIP/KIP family of cyclin inhibitory proteins, participates in inhibition of forming complexes that allow pRb to phosphorylate and lead the cell into mitosis. The expression of these important cell cycle regulator proteins was studied in a total of 96 non-Hodgkin's lymphoma (NHL) samples, which were classified according to the REAL classification. The expression of p27, pRb and the cell proliferation marker Ki-67 (MIB-1) was evaluated in lymphomas using immunohistochemistry. This study showed that there were coordinate changes in the expression of p27 and pRb in NHL. When compared to low-grade lymphomas, high-grade lymphomas showed significantly reduced expression of p27 and inversely pRb expression was increased ($P < 0.001$). Increase in expression of Ki-67 was parallel with pRb expression, and was mainly seen in cells that lacked p27 expression ($P < 0.0001$). This study suggests that changes in the control of the cell cycle closely relate to the pathobiology of NHL. © 2000 Cancer Research Campaign

**Keywords:** non-Hodgkin's lymphoma; p27 (Kip1); pRb; Ki-67 (MIB-1)

The non-Hodgkin's lymphomas (NHL) are a heterogeneous group of malignancies arising from B- or T-cell lymphatic systems. The current REAL classification of lymphomas is based on histology, immunohistochemistry and genetic changes (Harris et al 1994). Several regulators of the cell cycle are currently well known and their function and expression has been shown to be important in the initiation of cancer (Graña and Reddy, 1995; Hirama and Koeffler, 1995; Sherr, 1996). Because of their importance in the regulation of the cell cycle the presence and function of these regulator proteins in specific malignancies needs to be further studied.

The cell cycle is regulated by cyclins, cyclin-dependent kinases (CDK) and cyclin-CDK complex inhibitors. These inhibitory proteins can be divided into two classes differing in both sequence homology and their targets of inhibition (Hall et al, 1995). The CIP (cyclin inhibitory protein)/KIP(kinase inhibitory protein) gene family products (p21, p27 and p57) bind to cyclins, whereas INK4 gene family products (p15, p16, p18 and p19) bind to CDK4/6 (Graña and Reddy, 1995; Hall et al, 1995; Hirama and Koeffler, 1995; Sherr and Roberts, 1995; Sherr, 1996). Cyclin D and CDK form complexes able to phosphorylate pRb protein which functions in its hypophosphorylated form as a negative regulator of the cell cycle during $G_1$ phase. Cyclin D:CDK4/6 complexes phosphorylate retinoblastoma (RB) protein which then releases transcription factors, and allows cells to enter the S phase. Mutations of the RB gene or alterations in cyclin D:CDK complex inhibitory genes lead to loss of the principal function of the Rb product – growth control at $G_1$/S border.

The expression of cyclin-dependent kinase inhibitor p27/Kip1, a member of the p21 family, is high in cells inhibited by cell contact, by the cytokine transforming growth factor-β (TGF-β), or by serum deprivation (Polyak et al, 1994). The present evidence indicates that TGF-β induces cell cycle arrest through the cooperative action of p15 and p27, or p21 (Reynisdóttir et al, 1995).

The non-Hodgkin's lymphoma is a common malignancy, and its incidence is increasing in older populations (Zheng et al, 1992). Although NHL can be very invasive, treatments should not be started until the NHL has been classified according to, e.g. the REAL classification, since treatments vary considerably between the subgroups. The classification of lymphomas is difficult and the aggressiveness is variable within the same diagnosis. Therefore, specific markers are needed to evaluate the aggressiveness of the tumour and to improve the accuracy of classification. We have studied proliferation status and the expression of p27 and pRb, which are both cell cycle regulators involved in cyclin D-mediated responses, by immunohistochemical staining in non-Hodgkin's lymphomas.

### PATIENTS AND METHODS

The patient population included 96 non-Hodgkin's lymphoma (NHL) patients (age 16–90 years), who were treated at the Turku University Central Hospital for various histological subtypes of B-cell non-Hodgkin's lymphoma during years 1987–1996. The patient group was heterogeneous consisting of either high ($n = 52$) or low-grade ($n = 44$) malignant tumours. One representative nodal block from each case was retrieved for investigation. The

*Received 24 June 1999*
*Revised 26 June 2000*
*Accepted 12 July 2000*

*Correspondence to:* M Kiviniemi samples were reviewed and classified histologically according to the REAL classification (Harris et al, 1994). All immunohistochemistry was performed in Turku Central University Hospital laboratory of pathology, which has been certificated by the Finnish Medical Association, and is a member in an interlaboratory control programme (Labquality, Helsinki, Finland).

### Immunohistochemistry

The expression of cell cycle regulators p27, pRb and cell proliferation marker Ki-67 was investigated with immunohistochemistry as follows:

### *p27 and pRb*

The 96 tumour samples were fixed overnight with 10% neutral buffer formalin and otherwise treated with standard pathology laboratory methods used for routine lymphoma histopathology specimens. Paraffin embedded tumour samples were then cut in 5 µm thick sections on silane-coated glass slides. The sections were deparaffinized, rehydrated and treated for 5 min with 0.5% NP-40 (Sigma, St. Louis MO, USA) in TBS. For antigen retrieval the slides were treated in a microwave oven by boiling in 10 mM sodium citrate buffer (pH 6.0) for 10 min; after this, the slides were allowed to soak in the hot buffer for an additional 20 min. The tissue sections were then washed, blocked with normal serum and incubated overnight at 4°C with monoclonal mouse anti-human p27 antibody (Transduction Laboratories, Lexington, KY, USA) at a 1:1000 dilution or with monoclonal mouse anti-human pRb antibody (PharMingen, San Diego CA, USA) at a 1:500 dilution. The detection was performed with an anti-mouse ABC kit (Vector Laboratories, Burlingame CA, USA). After 2 washes in TBS, the samples were allowed to react with a biotinylated secondary antibody at a 1:200 dilution for 30 min at room temperature and ABC at a 1:75 dilution for 30 min at room temperature. The slides were stained with diaminobenzidine, washed, counterstained with Mayer's haematoxylin, dehydrated, treated with xylene and mounted with Mountex (Histolab Products AB, Sweden).

Both p27- and pRb-immunostainings were done in a single batch to avoid run-to-run variability. Samples of normal tonsil and retinoblastoma were included in each batch of stainings as controls. Control sections were also processed without the primary antibody to test the integrity of the staining procedure.

### *Ki-67*

Immunohistochemistry for 96 samples was performed with TechMate 500 (Dako, Denmark). From each of the selected blocks, a 5-µm tissue section was cut on a positively charged glass slide (ChemMate, DAKO A/S, Glostrup, Denmark). These sections were then deparaffinized with xylene, rehydrated through graded series of alcohol and washed three times with TBS. For antigen retrieval, the samples were treated for 10 min in a microwave oven in boiling 10 mM sodium citrate buffer, pH 6.0. After boiling the slides were allowed to soak in the hot buffer at room temperature for additional 20 min, and were then again washed with TBS. Immunohistochemistry was performed in an automated processor. Steps performed in the immunostainer include blocking with normal horse serum, application of primary antibody, application of secondary avidin-biotin-peroxidase-conjugated antibody, development with 3,3-diaminobenzidine as a substrate, and washes between each step. A light counterstaining with Mayer's haematoxylin was included. The primary mouse monoclonal Ki-67/MIB-1 antibody (Immunotech, Marseille, France) was applied for 27 min at a dilution of 1:200. All steps were performed in room temperature with standardized reaction times, allowing reliable comparison between the samples.

### Interpretation of staining

The interpretation and the evaluation of immunohistochemical staining results were performed blindly, and without reference to the clinical history of the patients. All the stainings were evaluated by two of the authors (MK and K-OS), and the classification is a concensus of these evaluations. The samples from the same tumour with different immunohistochemical stainings were evaluated concurrently. Tumour samples were graded according to their nuclear p27 and pRb staining into four groups (negative (0), low (1), intermediate (2) and high (3) expression). In negative samples staining was seen in benign cells, but not in malignant areas. In low and intermediate staining less or more than 50% of the tumour cells showed expression of p27 or pRb, respectively. Samples were graded into the high-expression group when 75% or more of the tumour cells were stained. Grading was based on the amount of stained cells in tumour area. Observations included only tumour cells excluding normal lymphocytes always present in lymphoid tumours. The normal lymphocytes and stromal cells provided internal positive and negative control in each sample. The few samples with no positive labelling in either tumour cells or benign cells were considered inconclusive and discarded from further analysis, as they could not be reliably verified as negative tumours due to the lack of internal positive control. The sample of retinoblastoma, which served as pRb-negative control, showed no staining for pRb but expressed p27 intensely. The section of tonsil representing normal lymphoid tissue showed marked p27 staining, while pRb expression was only seen in scattered cells in reactive centres. No positive staining was observed in these control sections when processed with exclusion of primary antibody. Since the evaluation was based only on the number of stained cells, any controls for levels of intensity were not in use. We chose to evaluate the most intense area of staining instead of random fields, because we believe that by assessing the area of highest proliferation we have analysed the most biologically active compartment of each tumour – the part which ultimately determines the prognosis of the patient and which also is the main target of any cancer therapy. In addition, there were usually no great differences in staining between different areas in tumours.

The tumour proliferating status was evaluated in Ki-67 (MIB-1) stained samples by counting the percentage of labelled cells. 500–600 cells were counted from the area of most intense staining in the sample. Cells were estimated as Ki-67 (MIB-1) positive if they showed any staining in the nucleus.

### Statistical analyses

Statistical analyses were performed using the SAS Release 6.12, GLM-procedure program. To evaluate the expressions of p27 and pRb against proliferating status of the samples (Ki-67 staining) one-way analyses of variance (ANOVA) was computed. p27 and pRb immunostainings were correlated with histological grade (high-grade/low-grade) and subtype of lymphomas using Chi Square test. The level of significance was set at $P < 0.05$.

© 2000 Cancer Research Campaign

**Table 1** Relationship between p27 immunostaining and REAL classification

| Intensity of staining | Number of tumours (%) SLBCL (*n* = 15) | FCL (*n* = 29) | MCL (*n* = 19) | DLBCL (*n* = 33) |
|---|---|---|---|---|
| 0 | 0 (0) | 2 (7) | 3 (16) | 19 (58) |
| 1 | 0 (0) | 2 (7) | 4 (21) | 12 (36) |
| 2 | 1 (7) | 6 (20) | 9 (47) | 2 (6) |
| 3 | 14 (93) | 19 (66) | 3 (16) | 0 (0) |

SLBCL = small lymphocytic B-cell lymphoma; FCL = follicular-centre lymphoma; MCL = mantle-cell lymphoma; DLBCL = diffuse large B-cell lymphoma

**Table 2** Relationship between pRb immunostaining and REAL classification

| Intensity of staining | Number of tumours (%) SLBCL (*n* = 15) | FCL (*n* = 29) | MCL (*n* = 19) | DLBCL (*n* = 33) |
|---|---|---|---|---|
| 0 | 12 (80) | 8 (28) | 6 (32) | 2 (6) |
| 1 | 3 (20) | 16 (55) | 4 (21) | 4 (12) |
| 2 | 0 (0) | 5 (17) | 1 (5) | 11 (33) |
| 3 | 0 (0) | 0 (0) | 8 (42) | 16 (49) |

SLBCL = small lymphocytic B-cell lymphoma; FCL = follicular-centre lymphoma; MCL = mantle-cell lymphoma; DLBCL = diffuse large B-cell lymphoma

### RESULTS

Low-grade malignancies (small lymphocytic B-cell and follicular centre) showed intermediate or high expression of p27 in most cases (40/44, 91%) (Table 1). Small lymphocytic B-cell lymphoma samples showed a diffuse staining pattern throughout the sample (15/15, 100%) (Figure 1A), whereas follicle centre lymphomas showed marked p27 expression in the benign areas around follicles and benign cells in follicular areas (25/29, 86%) (Figures 1C and 1E).

High-grade malignancies showed more variation in p27 staining (Table 1). Most high-grade malignancies (mantle cell and diffuse large B-cell lymphoma) were negative or showed only very low staining of p27 (38/52, 73%). In the group of most common high-grade malignancy, diffuse large B-cell lymphoma, only two samples showed considerable expression for p27 (2/33, 6%) (Figure 1G). The mantle cell lymphomas showed a very heterogeneous staining for p27 and were divided into all categories.

In most low-grade lymphomas the expression of pRb was weak (39/44, 89%) (Table 2). The model of staining in small lymphocytic B-cell lymphomas was high expression of p27 and negative expression of pRb in all of the samples (15/15, 100%) (Figure 1B) whereas follicle centre lymphomas showed high expression of p27 but low or negative expression of pRb (23/29, 79%). In follicular centre lymphomas marked pRb staining was only seen in follicle areas, when benign tissue around follicles remained negative (Figures 1D and 1F).

In tumour samples of diffuse large B-cell lymphomas pRb staining was intense (31/40, 78%) (Figure 1H) (Table 2). In the group of mantle-cell lymphomas stainings divided into all categories as with p27 immunostaining. However, in mantle-cell lymphoma samples with low p27 expression, pRb showed high expression (7/7) whereas when pRb was lost, marked expression of p27 was present (10/12, 83%). The inverse correlation between p27 and pRb stainings in individual tumours can be clearly seen in Tables 3 and 4.

The proliferative status of tumour samples investigated with Ki-67 (MIB-1) immunostaining was low in low-grade and high in high-grade malignant tumours. The percentage of proliferating cells analysed by Ki-67 (MIB-1) immunostaining increased as p27 expression was decreased ($P < 0.0001$, F = 12.01, ANOVA df = 3). The opposite was seen when correlating pRb against Ki-67 ($P < 0.0001$, F = 13.98, ANOVA df = 3) (Figure 2).

The comparison between low-grade malignancies (small lymphocytic and follicular centre lymphomas, $n = 44$) and high-grade malignancies (diffuse large B-cell and mantle cell lymphomas, $n = 52$) revealed that low-grade malignancies showed higher expression of p27 than high-grade malignancies ($P < 0.001$, Chi-square test, df = 3), and the pRb staining was dominant in high-grade malignant samples ($P < 0.001$, Chi-square test, df = 3). When comparing p27 and pRb expressions to histological grading and proliferative activity (Ki-67 expression), coordinate changes were observed in all subtypes of NHL ($P < 0.05$).

**Table 3** Inverse correlation between p27 and pRb expression in individual tumour samples. Tumours with negative (0) or low (1) expression of p27 show marked expression of pRb, and vice versa

| p27 | 0 | 1 | 2 | 3 |
|---|---|---|---|---|
| pRB | | | | |
| 0 | 2 | 0 | 7 | 19 |
| 1 | 2 | 3 | 6 | 16 |
| 2 | 7 | 6 | 3 | 1 |
| 3 | 13 | 9 | 2 | 0 |

### DISCUSSION

This study concentrated in observing changes in the most common forms of lymphoma, i.e. diffuse large B-cell, mantle-cell, follicular centre and small lymphocytic B-cell lymphoma, which in its subsequent course is called chronic lymphocytic B-cell leukaemia. The rarity of some lymphoma entities slows effective research in these subgroups, e.g. the mantle-cell lymphoma, of which incidence has increased recently. This could be due to more accurate diagnostics using REAL classification. Clinical trials have failed to significantly improve the outcome of patients with mantle-cell lymphoma, and the disease leads to death in approximately 3 years (Non-Hodgkin's Lymphoma Classification Project, 1997). In the present study the group of mantle-cell lymphomas produced the most conflicting staining pattern. Although tumour samples showed either marked p27 or pRb expression, they differed from both low- and high-grade lymphoma groups according to their stainings and therefore could not be characterized into either of these groups in this study.

The role of cell cycle regulators has been a focus of cancer research, and understanding of the initiation of cancer has increased rapidly. In haematological malignancies several $G_1$/S checkpoint inhibitors have recently been under investigation. The role of p16 gene alterations has been widely described, e.g. in adult T-cell leukaemia and lymphoma (Uchida et al, 1998) and both B- and T-cell lymphoid cell lines (Stranks et al, 1995). A recent immunohistochemical study of p16 and pRb expressions in non-Hodgkin's lymphomas concluded a loss of these two proteins in

© 2000 Cancer Research Campaign

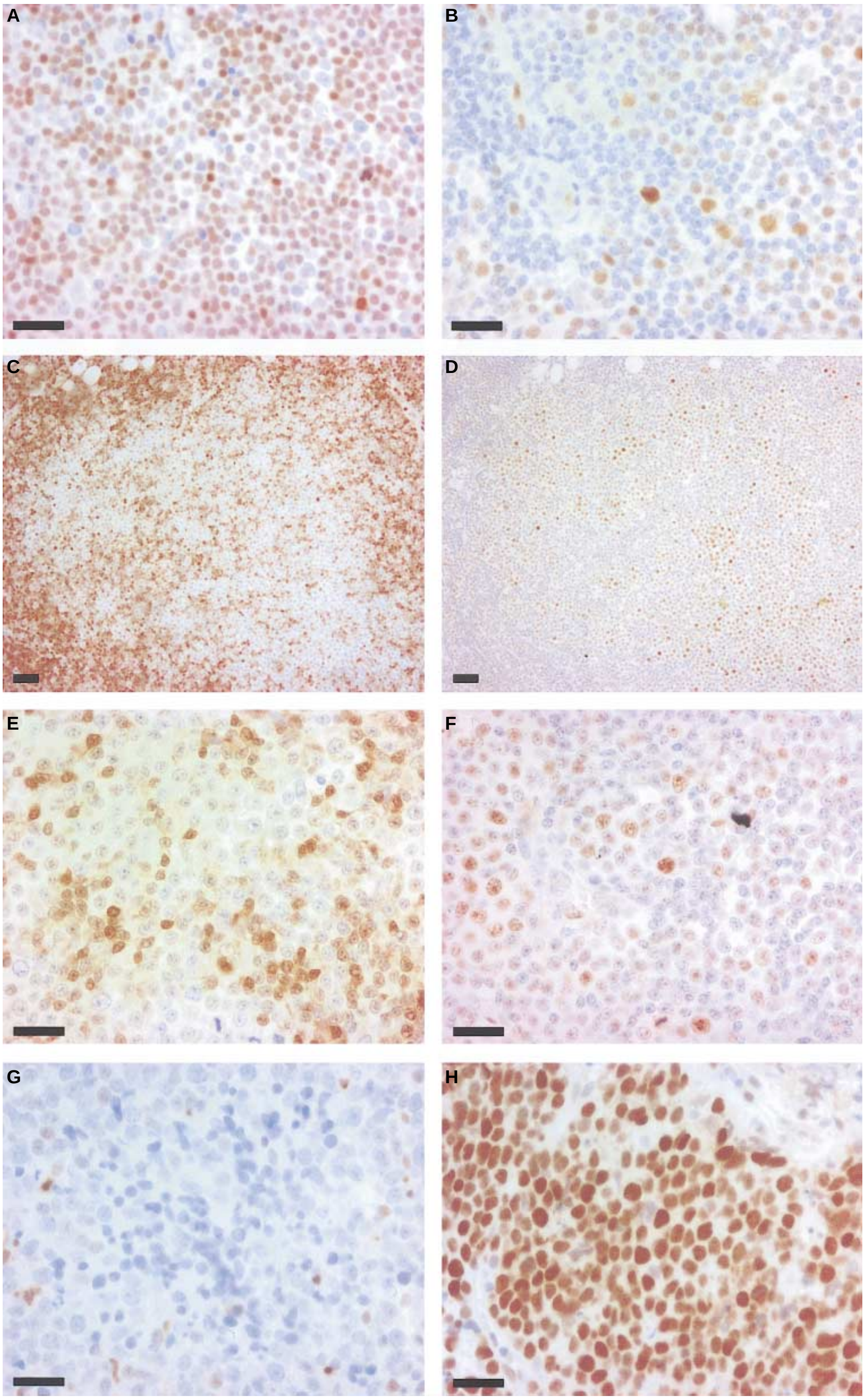

**Figure 1** All the stainings are counterstained with haematoxylin, the scale bar in the pictures is 25 µm. Panels **A**, **C**, **E** and **G** immunohistochemically stained for p27, panels **B**, **D**, **F** and **H** immunohistochemically stained for pRb. (**A**) A photomicrograph of a small lymphocytic lymphoma, case number 11. Almost all lymphoma cells are strongly stained for p27. The blast cells are negative. (**B**) The Rb expresses only in blast cells in small lymphocytic lymphoma. (**C**) All benign areas of follicle-centre lymphoma, case number 20, are strongly stained, note the high expression of p27 in mantle zone. (**D**) Inversely stained follicle of follicular-centre lymphoma as compared to p27 staining. (**E**) A closer view of the same field of the follicular-centre lymphoma as seen in Figure 2. Normal lymphocytes are p27-positive. (**F**) A closer view of the follicle in follicular-centre lymphoma showing strong pRb staining in blast cells. Note the positively stained mitoses in the bottom of the field. (**G**) A diffuse large B-cell lymphoma, case number 95. Only normal lymphocytes show any p27 expression. (**H**) A strong expression of pRb is seen in diffuse large B-cell lymphoma

 *© 2000 Cancer Research Campaign*

**Table 4** p27, pRb and Ki-67 protein expression in individual tumours investigated by immunohistochemistry.

| Case (low-grade) | Protein expression by immunohistochemistry p27 | pRb | Ki-67(%) | Case (high-grade) | Protein expression by immunohistochemistry p27 | pRb | Ki67(%) |
|---|---|---|---|---|---|---|---|
| 1 SLBCL | +++ | – | 1 | 45 MCL | ++ | – | 0 |
| 2 SLBCL | +++ | – | 1 | 46 MCL | + | +++ | 0 |
| 3 SLBCL | ++ | + | 1 | 47 MCL | +++ | – | 1 |
| 4 SLBCL | +++ | – | 5 | 48 MCL | +++ | + | 5 |
| 5 SLBCL | +++ | – | 10 | 49 MCL | ++ | – | 10 |
| 6 SLBCL | +++ | + | 10 | 50 MCL | +++ | + | 10 |
| 7 SLBCL | +++ | – | 10 | 51 MCL | ++ | + | 10 |
| 8 SLBCL | +++ | – | 10 | 52 MCL | ++ | – | 20 |
| 9 SLBCL | +++ | – | 10 | 53 MCL | ++ | – | 20 |
| 10 SLBCL | +++ | – | 15 | 54 MCL | + | +++ | 25 |
| 11 SLBCL | +++ | – | 15 | 55 MCL | + | +++ | 30 |
| 12 SLBCL | +++ | + | 20 | 56 MCL | ++ | + | 35 |
| 13 SLBCL | +++ | – | 25 | 57 MCL | – | +++ | 40 |
| 14 SLBCL | +++ | – | 30 | 58 MCL | ++ | – | 40 |
| 15 SLBCL | +++ | – | 50 | 59 MCL | ++ | +++ | 50 |
| 16 FCL | +++ | + | 1 | 60 MCL | ++ | ++ | 50 |
| 17 FCL | +++ | – | 5 | 61 MCL | + | +++ | 60 |
| 18 FCL | ++ | ++ | 10 | 62 MCL | – | +++ | 80 |
| 19 FCL | +++ | ++ | 10 | 63 MCL | – | +++ | 95 |
| 20 FCL | +++ | – | 10 | 64 DLBCL | – | – | 0 |
| 21 FCL | + | ++ | 10 | 65 DLBCL | + | ++ | 1 |
| 22 FCL | ++ | – | 10 | 66 DLBCL | + | + | 5 |
| 23 FCL | +++ | – | 10 | 67 DLBCL | – | +++ | 5 |
| 24 FCL | +++ | – | 10 | 68 DLBCL | – | ++ | 5 |
| 25 FCL | +++ | + | 10 | 69 DLBCL | – | +++ | 20 |
| 26 FCL | +++ | – | 10 | 70 DLBCL | ++ | +++ | 20 |
| 27 FCL | +++ | + | 10 | 71 DLBCL | – | – | 20 |
| 28 FCL | +++ | + | 15 | 72 DLBCL | – | ++ | 20 |
| 29 FCL | +++ | + | 20 | 73 DLBCL | + | ++ | 25 |
| 30 FCL | ++ | + | 20 | 74 DLBCL | – | +++ | 25 |
| 31 FCL | ++ | + | 20 | 75 DLBCL | – | ++ | 25 |
| 32 FCL | + | + | 20 | 76 DLBCL | – | + | 30 |
| 33 FCL | +++ | + | 20 | 77 DLBCL | + | +++ | 30 |
| 34 FCL | +++ | + | 20 | 78 DLBCL | – | +++ | 30 |
| 35 FCL | +++ | + | 24 | 79 DLBCL | + | ++ | 35 |
| 36 FCL | +++ | + | 30 | 80 DLBCL | – | +++ | 40 |
| 37 FCL | – | ++ | 30 | 81 DLBCL | + | ++ | 40 |
| 38 FCL | +++ | + | 30 | 82 DLBCL | + | +++ | 50 |
| 39 FCL | +++ | – | 30 | 83 DLBCL | + | + | 60 |
| 40 FCL | ++ | – | 30 | 84 DLBCL | – | +++ | 70 |
| 41 FCL | +++ | + | 40 | 85 DLBCL | ++ | ++ | 70 |
| 42 FCL | ++ | + | 40 | 86 DLBCL | + | +++ | 75 |
| 43 FCL | – | ++ | 45 | 87 DLBCL | – | +++ | 80 |
| 44 FCL | +++ | + | 55 | 88 DLBCL | + | ++ | 80 |
| | | | | 89 DLBCL | – | +++ | 80 |
| | | | | 90 DLBCL | – | +++ | 85 |
| | | | | 91 DLBCL | – | ++ | 85 |
| | | | | 92 DLBCL | + | +++ | 90 |
| | | | | 93 DLBCL | – | ++ | 90 |
| | | | | 94 DLBCL | – | + | 90 |
| | | | | 95 DLBCL | – | +++ | 95 |
| | | | | 96 DLBCL | + | +++ | 95 |

SLBCL = small lymphocytic B-cell lymphoma; FCL = follicular-centre lymphoma; MCL = mantle-cell lymphoma; DLBCL = diffuse large B-cell lymphoma; (–) negative staining in malignant cells; (+) protein expression in less than 50% of malignant cells; (++) protein expression in 50–75% of malignant cells; (+++) more than 75% of malignant cells show protein expression. Ki-67 expression in percentage of 500–600 counted cells high-grade malignant lymphomas but not in low-grade malignancies (Geradts et al, 1998). The explanation for variance in our results when compared to other studies investigating p27, pRb and other cell cycle regulator proteins relates to differences in scoring methods. While some groups (Geradts et al, 1998) have only graded tumours to positive or negative, we chose to grade tumours to four groups to increase the accuracy of evaluation. Also, in some studies a larger part of the tumour has been graded, while we concentrated the evaluation in the most intensely stained area, seeking the most biologically active area of the tumour. In immunohistochemical studies one also has to be critical for the sensitiveness of the method. Some antibodies produce cytoplasmic background, and it may interfere with reliable detection of nuclear signals. The antibodies used in our study show only distinct

© 2000 Cancer Research Campaign

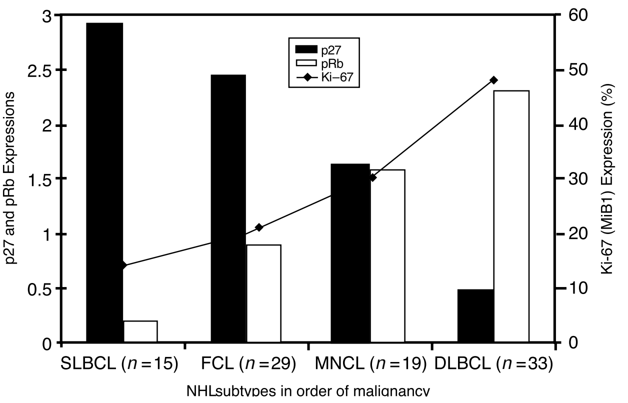

**Figure 2** The expression of Ki-67 (MIB-1) increases parallel to pRb and inversely to p27 expressions. Stainings of p27 and pRb are presented as an average of immunohistochemical staining scores in each subgroup (see also Tables 3 and 4). Abbreviations and standard deviations in the Figure: SLBCL = small lymphocytic B-cell lymphoma (SD p27 0.26, pRb 0.41), FCL = follicular-centre lymphoma (SD p27 0.91, pRb 0.67), MCL = mantle cell lymphoma (SD p27 0.95, pRb 1.35), DLBCL = diffuse large B-cell lymphoma (SD p27 0.62, pRb 0.90)

nuclear staining, which was used as a basis of scoring. Nevertheless, the comparison between different studies is possible when one combines negative and low expressions to one group and intermediate and high expressions to another group. Therefore we claim, that the present study provides new knowledge with a wider perspective of the $G_1$/S checkpoint control in NHL.

Ki-67 (MIB-1) immunostaining has been commonly used as a proliferation marker, and it is therefore appropriate as a correlation against new molecular markers. Proliferative activity measured with Ki-67 has been shown to act as a significant predictor of survival in NHL (Mochen et al, 1997; Korkolopoulou et al, 1998). p27 expression has previously been described to inversely relate to the proliferation index measured by Ki-67 in mantle-cell lymphomas (Quintanilla-Martinez et al, 1998), whereas MALT-NHLs have showed parallel expression for Ki-67 and pRb (Stefanaki et al, 1998). Studies correlating the expression of Ki-67, p27 and pRb in the same lymphomas have not been reported previously.

The results of p27 expression in correlation to tumour prognosis have been conflicting in different malignancies. Decreased nuclear expression of p27 has been shown to be a significant predictor of poor survival in breast cancer (Catzavelos et al, 1997; Porter et al, 1997) and colorectal cancer (Yasui et al, 1997), whereas high levels of p27 were associated with a shorter survival in chronic B-cell lymphocytic leukaemia (Vrhovac et al, 1998). Homozygous deletions or point mutations of p27 have been observed in adult T-cell leukaemias and lymphomas (Hatta et al, 1997). The present study revealed a significant correlation between p27 expression and the grade and proliferative status of tumour. The presence of p27 protein, which functions as a cell cycle inhibitor, indicated lower proliferative rate, which was characteristic of low-grade malignancies. Loss of cell cycle inhibition as revealed by higher percentage of Ki-67-positive cells and increased proliferative rate was observed in most high-grade tumours, whereas p27 immunostaining was low or undetectable among them. In low-grade lymphomas p27 staining showed a marked resemblance to benign cells.

Mutations or loss of expression of pRb has been described to be a cause of $G_1$/S-checkpoint disruption in all common cancers. NHL subtypes can be roughly arranged in linear order from the lowest to the highest grade of malignancy (Harris et al, 1994; Howard and Shipp, 1998; Zucca et al, 1998). When observing this order of lymphomas, in the present study increase in both pRb and Ki-67 expression from low- to high-grade malignancies was seen. All samples of small lymphocytic lymphoma showed low expression of pRb, or were completely negative. This may be explained by the fact that the malignant cells in small lymphocytic lymphoma behave close to normal lymphocytes, which also have low pRb expression. Similarly to our findings, there are recent reports of high levels of pRb in high-grade malignant lymphomas (Geradts et al, 1998; Cinti et al, 2000).

The main function of pRb is to act as a monitor of cell cycle progression. pRb is phosphorylated by cyclin-D:CDK-complexes at late $G_1$ phase, and further phosphorylation is provided by other cyclins until dephosphorylation after mitosis. We are convinced that the immunohistochemically detected pRb represents both hypo- and hyperphosphorylated protein, as even mitotic cells stain positively (Figure 1F), and we have observed a positive correlation between pRb expression and Ki-67 staining also in benign proliferating tissues, such as skin, placenta and endometrium (Sauroja et al, unpublished). In lymphatic B cells, pRb may be needed for terminal differentation, as in $pRb_{-/-}$ mice haemopoietic lineages fail to differentiate (Clarke et al, 1992; Lee et al, 1992). On the other hand, in lymphatic malignancies the growth-suppressive action of pRb may be overcome by overexpression of cyclin D, which has been shown in B-cell lymphomas due to translocation t(11;14)(q13;q32) of the cyclin D gene (Howard and Shipp, 1998). The regulation of cell cycle progression by pRb can also be disrupted by loss of control of cyclin: CDK-complexes by either p16 or p21 families of cyclin-CDK-inhibitors, such as the loss of p27 expression shown in the present study. Furthermore, while the $G_1$/S regulation by pRb is mediated by binding to E2F family of transcription factors, later in the cell cycle pRb can be bound by oncoproteins like c-Myc, c-Abl, and MDM2 (Rustgi et al, 1991; Welch and Wang, 1993; Xiao et al, 1995). Considering all these mechanisms in the pRb pathway, our finding of the apparently paradoxical correlation between pRb expression and cell proliferation (Ki-67 expression) seems only rational. Although it would be expected that tumours with high levels of pRb had a reduced proliferative rate, observations similar to ours have been reported recently (Leoncini et al, 1999; Cinti et al, 2000).

In conclusion, this study showed coordinate changes in expression of p27 and pRb in non-Hodgkin's lymphomas. The results indicate that the differences in expression of p27 and pRb may improve the evaluating of the aggressiveness of lymphomas. Immunochemical stainings for p27 and pRb appeared to be useful in differentiating between low- and high-grade lymphomas and therefore may be suitable as tools for the lymphoma pathologist. High-grade lymphomas expressed p27 significantly less than low-grade malignancies, whereas the expression of pRb was dominant in high-grade malignancies when the p27 control was lost. Changes in control of cell cycle appeared to be closely related to the pathobiology of non-Hodgkin's lymphomas. Further studies are needed to investigate whether prognostic significance of p27 and pRb expression can be found within specific non-Hodgkin's lymphoma types.

 © 2000 Cancer Research Campaign

### ACKNOWLEDGEMENTS

This work was funded by The Cancer Society of Southwest Finland, The Turku Graduate School of Biomedical Sciences and the EVO-grants of Turku University Central Hospital. Docent Seija Grenman is acknowledged for reviewing the article.

### REFERENCES

Catzavelos C, Bhattacharya N, Ung VC, Wilson JA, Roncari L, Sandhu C, Shaw P, Yeger H, Morava-Prozner I, Kapusta L, Franssen E, Pritchard KI and Slingerland JM (1997) Decreased levels of the cell-cycle inhibitor $p27^{Kip1}$ protein: Prognostic implications in primary breast cancer. *Nat Med* **2**: 227–230

Cinti C, Leoncini L, Nyongo A, Ferrari F, Lazzi S, Bellan C, Vatti R, Zamparelli A, Cevenini G, Tosi GM, Claudio PP, Maraldi NM, Tosi P and Giordano A (2000) Genetic alterations of the retinoblastoma-related gene RB2/p130 identify different pathogenetic mechanisms in and among Burkitt's lymphoma subtypes. *Am J Pathol* **156**: 751–760

Clarke AR, Maandag ER, van Roon M, van der Luft NM, van der Valk M, Hooper ML, Berns A and te Riele H (1992) Requirement for a functional Rb-1 gene in murine development. *Nature* **359**: 328–330

Geradts J, Andriko JW and Abbondanzo SL (1998) Loss of tumor suppressor gene expression in high-grade but not low-grade non-Hodgkin's lymphomas. *Am J Clin Pathol* **109**: 669–674

Graña X and Reddy EP (1995) Cell cycle control in mammalian cells: role of cyclins, cyclin-dependent kinases (CDKs), growth suppressor genes and cyclin-dependent kinase inhibitors (CKIs). *Oncogene* **11**: 211–219

Hall M, Bates S and Peters G (1995) Evidence for different modes of action of cyclin-dependent kinase inhibitors: p15 and p16 bind to kinases, p21 and p27 bind to cyclins. *Oncogene* **11**: 1581–1588

Harris NL, Jaffe ES, Stein H, Banks PM, Chan JKC, Cleary ML, Delso G, De Wolf-Peeters C, Falini B, Gatter KC, Grogan TM, Isaacson PG, Knowless DM, Mason DY, Muller-Hermelink HK, Pileri SA, Piris MA, Ralfkiaer E and Waroke RA (1994) A revised European–American classification of lymphoid neoplasms: a proposal from the International Lymphoma Study Group. *Blood* **84**: 1361–1392

Hirama T and Koeffler HP (1995) Role of the cyclin-dependent kinase inhibitors in the development of cancer. *Blood* **86**: 841–854

Hatta Y, Yamada Y, Tomonaga M and Koeffler HP (1997) Extensive analysis of the retinoblastoma gene in adult T cell leukemia/lymphoma. *Leukemia* **11**: 984–989

Howard MO and Shipp MA (1998) The cellular and molecular heterogeneity of the aggressive non-Hodgkin's lymphomas. *Curr Opin Oncol* **10**: 385–391

Korkolopoulou P, Angelopoulou MK, Kontopidou F, Tsenga A, Patsouris E, Thomas-Tsagli E, Kittas C and Pangalis GA (1998) Prognostic relevance of apoptotic cell death in non-Hodgkin's lymphomas: a multivariate survival analysis including Ki67 and p53 oncoprotein expression. *Histopathology* **33**: 240–247

Lee EY, Chang CY, Hu N, Wang YC, Lai CC, Herrup K, Lee WH and Bradley A (1992) Mice deficient for Rb are nonviable and show defects in neurogenesis and haematopoiesis. *Nature* **359**: 288–294

Leoncini L, Bellan C, Cossu A, Claudio PP, Lazzi S, Cinti C, Cevenini G, Megha T, Laurini L, Luzi P, Orcioni GF, Piccioli M, Pileri S, Giardino C, Tosi P and Giordano A (1999) Retinoblastoma-related p107 and pRb2/p130 proteins in malignant lymphomas: distinct mechanisms of cell growth control. *Clin Cancer Res* **5**: 4065–4072

Mochen C, Giardini R, Costa A and Silvestrini R (1997) MiB1 and S-phase cell fraction predict survival in non-Hodgkin's lymphomas. *Cell Prolif* **1**: 37–47

Non-Hodgkin's Lymphoma Classification Project (1997) A clinical evaluation of the International Lymphoma Study Group classification of non-Hodgkin's lymphoma. *Blood* **89**: 3909–3918

Polyak K, Kato J, Solomon MJ, Sherr CJ, Massague J, Roberts JM and Koff A (1994) $p27^{Kip1}$, a Cyclin-Cdk inhibitor, links transforming growth factor-β and contact inhibition to cell cycle arrest. *Genes Dev* **8**: 9–22

Porter PL, Malone KE, Heagerty PJ, Alexander GM, Gatti LA, Firpo EJ, Daling JR and Roberts JM (1997) Expression of cell-cycle regulators $p27^{Kip1}$ and cyclin E, alone and in combination, correlate with survival in young breast cancer patients. *Nat Med* **3**: 222–225

Quintanilla-Martinez L, Thiemblemont C, Fend F, Kumar S, Pinyol M, Campo E, Jaffe ES and Raffeld M (1998) Mantle cell lymphomas lack expression of p27Kip1, a cyclin-dependent kinase inhibitor. *Am J Pathol* **153**: 175–182

Reynisdóttir I, Polyak K, Iavarone A and Massagué J (1995) Kip/Cip and Ink4 Cdk inhibitors cooperate to induce cell cycle arrest in response to TGF-β. *Genes Dev* **9**: 1831–1845

Rustgi AK, Dyson N and Bernards R (1991) Amino-terminal domains of c-myc and N-myc proteins mediate binding to the retinoblastoma gene product. *Nature* **352**: 541–544

Sherr CJ (1996) Cancer cell cycles. *Science* **274**: 1672–1677

Sherr CJ and Roberts JM (1995) Inhibitors of mammalian $G_1$ cyclin-dependent kinases. *Genes Dev* **9**: 1149–1163

Stefanaki K, Tzardi M, Kouvidou C, Chaniotis V, Bolioti M, Vlychou M, Zois M, Kakolyris S, Delides G, Rontogianni D, Georgoulias V and Kanavaros P (1998) Expression of p53, p21, mdm2, Rb, bax and Ki-67 proteins in lymphomas of the mucosa-associated lymphoid (MALT) tissue. *Anticancer Res* **18**: 2403–2408

Stranks G, Height SE, Mitchell P, Jadayel D, Yuille MA, De Lord C, Clutterbuck RD, Treleaven JG, Powles RL and Nacheva E (1995) Deletions and rearrangement of CDKN2 in lymphoid malignancy. *Blood* **35**: 893–901

Uchida T, Kinoshita T, Murate T, Saito H and Hotta T (1998) CDKN2 ($MTS1/p16^{INK4A}$) gene alterations in adult T-cell leukemia/lymphoma. *Leuk Lymphoma* **29**: 27–35

Vrhovac R, Delmer A, Tang R, Marie JP, Zittoun R and Ajchenbaum-Cymbalista F (1998) Prognostic significance of the cell cycle inhibitor $p27^{Kip1}$ in chronic B-cell lymphocytic leukemia. *Blood* **91**: 4694–4700

Welch PJ and Wang JY (1993) A C-terminal protein-binding domain in the retinoblastoma protein regulates nuclear c-Abl tyrosine kinase in the cell cycle. *Cell* **75**: 779–790

Xiao ZX, Chen J, Levine AJ, Modjtahedi N, Xing J, Sellers WR and Livingston DM (1995) Interaction between the retinoblastoma protein and the oncoprotein MDM2. *Nature* **375**: 694–698

Yasui W, Kudo Y, Semba S, Yokozaki H and Tahara E (1997) Reduced expression of cyclin-dependent kinase inhibitor $p27^{Kip1}$ is associated with advanced stage and invasiveness of gastric carcinomas. *Jpn J Cancer Res* **88**: 625–629

Zheng T, Mayne ST, Boyle P, Holford TR, Liu WL and Flannery J (1992) Epidemiology of non-Hodgkin's lymphomas in Connecticut. *Cancer* **70**: 840–849

Zucca E, Bertoni F, Roggero E and Cavalli F (1998) Management of rare forms of lymphoma. *Curr Opin Oncol* **10**: 377–384

© 2000 Cancer Research Campaign
